# Why scapegoating can ruin an apology: The mediated-moderation model of appropriate crisis response messages in the context of South Korea

**DOI:** 10.3389/fpsyg.2022.1082152

**Published:** 2023-01-16

**Authors:** Sungbin Youk, Hee Sun Park

**Affiliations:** ^1^Department of Communication, University of California, Santa Barbara, Santa Barbara, CA, United States; ^2^School of Media and Communication, Korea University, Seoul, Republic of Korea

**Keywords:** apology, communicative responsibility, crisis communication, process analysis, scapegoating, South Korea

## Abstract

**Introduction:**

As South Korean companies frequently use apologies for various crisis situations and pair them with other types of crisis response strategies (i.e., scapegoating), theory-driven recommendations for crisis response messages may fall short in practice. This study empirically examines the effectiveness of two crisis response messages (i.e., apology + compensation vs. apology + scapegoating) by integrating the theory of communicative responsibility and situational crisis communication theory.

**Methods:**

South Korean participants (*n* = 392) read one of two vignettes: the vignettes described an automobile company’s apology for malfunctioning seat belts which included either compensation or scapegoating. The participant’s perceived communicative responsibility, appropriateness of the apology, and reputation of the company were measured. Process analysis was conducted to examine the mediated-moderation effect of the crisis response messages.

**Results and Discussion:**

The findings indicate that an apology that is provided with compensation is more appropriate than those with scapegoating. The appropriateness is moderated by the perceived symmetry in communicative responsibility, and fully mediates the relationship between apology type and reputation. This study integrates two theoretical models to examine the mechanism behind the crisis response strategies from the perspective of the message receivers, while considering the cultural and normative context of South Korea.

## Introduction

A crisis is an unpredictable threat to stakeholders, which affects an organization’s reputation and performance. To advance an effective response to a crisis, Situational Crisis Communication Theory (SCCT; Coombs and Holladay, 2002; Coombs, 2007a) recommends the organizations to first evaluate the crisis, understand their responsibility for the crisis, and then devise a response accordingly. For instance, the theory recommends the organization to use apologies when it is highly responsible for the crisis (Choi and Chung, 2013; Ma and Zhan, 2016). Although the theory provides a framework that can be applied to better understand the nature of different crisis situations, the theory-driven recommendation may fall short in practice when the cultural and normative factors, as well as the variations within the content of a crisis response message are overlooked.

In South Korea, organizations use apologies inconsistently with SCCT. First, organizations frequently included apologies in their crisis response messages regardless of the nature of the crisis (Park and Ha, 2014). It is normative for South Koreans to apologize to the listener even when the listener has wronged them (Barnlund and Yoshioka, 1990; Lee and Park, 2011). Additionally, Koreans are likely to evaluate apologies more positively and emulate others’ apologies (Park et al., 2005; An et al., 2010). Therefore, when the consumers of a company positively evaluate its apology, other companies may subsequently model the message, giving rise to the prevalent amount of apologies used by South Korean companies.

Second, apologies vary in their content. According to SCCT, an apology needs to ask for forgiveness, express regret, show concern, reassure the prevention of a future crisis, and fully accept responsibility for the current crisis (Coombs et al., 2010). However, organizations may attempt to shift the blame and avoid compensating the victims of the crisis while apologizing (Dulaney and Gunn, 2018). The organizations attempt to take advantage of what the word ‘sorry’ may imply, while excluding any explicit acknowledgement of the crisis responsibility. The content of crisis response messages, especially apology, also intertwines with the cultural context: South Korean companies are less likely to give excuses compared to American companies but are more likely to provide compensation along with their apologies (Kim and Lee, 2021).

In addition to the discrepancy in theory and practice, SCCT falls short in explaining why certain crisis response messages are effective from the perspective of consumer psychology. According to the theory of communicative responsibility (CRT; Aune et al., 2005), consumers expect the company to have the responsibility for helping them understand the crisis situation. They will be unsatisfied with the crisis response message when it does not provide enough explanation and information about the crisis to the extent that they expect. Therefore, there are two different responsibilities a company has at the time of a crisis: responsibility for the crisis and responsibility for informing the consumers about the crisis.

This study examined the effect of an apology by surveying 392 Koreans using a set of validated vignettes. Following the blueprints of SCCT, the study hypothesized that the perceived appropriateness of the apology mediates the relationship between the types of apology (i.e., an apology with scapegoating vs. an apology with financial compensation) and the consumer’s perception of the company’s reputation. According to CRT, the perceived appropriateness of apologies will vary depending on how much the consumers expect the company to be responsible for informing them about the crisis. The mediated-moderation effect is tested using the process analysis (Hayes, 2018). From this study, we aim to stretch the boundaries of SCCT by (a) investigating crisis communication in a non-Western culture, (b) examining the variations and complexities in apologies, and (c) integrating CRT and SCCT to better understand the psychological state of the consumers, who are the receivers of the crisis response messages from the company.

### SCCT

SCCT, proposed by Coombs (2000), is a widely-used theoretical framework in crisis communication literature (Nwogwugwu, 2018). It explains which crisis response strategy is effective for restoring the organization’s reputation that was damaged from the crisis (Coombs and Holladay, 2002). The theory categorizes crises into one of three clusters depending on the level of the organization’s responsibility (Coombs, 2007b). The organization is less accountable for a victim crisis (e.g., natural disasters, rumors, and product tampering) as the crisis was unexpected, unforeseeable, and unintended. The organization is moderately responsible for an accidental crisis, which includes a product malfunction due to a technical error. When the organization is highly responsible for a crisis, it is categorized as a preventable crisis. For instance, organizational misdeeds, management misconduct, and human-lead recalls and breakdowns happen as a direct consequence of the organization’s actions and choices. The meta-analysis conducted by Ma and Zhan (2016) empirically validates these three crisis types: the reputation of a company decreases as it is more responsible for a crisis (*r* = −0.54).

To mitigate the negative impact of a crisis, SCCT recommends the organization to formulate appropriate crisis response strategies (Coombs, 2006, 2007a). The organizations should use response strategies that convey more acceptance of the crisis responsibility and concern for stakeholders when it is more responsible for the crisis (Coombs and Holladay, 2005). Like the three types of crises, the theory categorizes response strategies into three groups. Deny strategy (which is appropriate for the victim crisis) expresses the least amount of consideration and acceptance. The organization uses the deny strategy, such as attacking the accuser and blaming another party for the crisis (i.e., scapegoating), to reduce the attributed crisis responsibility (Coombs, 2007a). This strategy conveys the organization’s defensive position. Compared to the deny strategy, the diminish strategy (which is appropriate for the accidental crisis) conveys a moderate amount of concern and acceptance by arguing that the crisis was uncontrollable, or its damage is not as detrimental as it seems (e.g., giving excuses for the crisis; Coombs, 2007b). The rebuilding strategy (which is appropriate for the preventable crisis) provides materials and symbolic forms of aid to the crisis victims. For example, the organization may take full responsibility for a crisis by giving compensation and a sincere apology. Ma and Zhan’s meta-analysis (2016) found that response strategies were more effective when they were used consistently with the SCCT recommendation (*r* = 0.23).

### Cultural context of South Korea

Despite the popularity and usefulness of SCCT, its application to a wider, non-Western, global context is often overlooked (Coombs et al., 2010; Coombs and Laufer, 2018; Nwogwugwu, 2018). The effectiveness of certain response strategies as recommended by SCCT may vary across cultures due to the communicative norms (Guan et al., 2009; Lee and Park, 2011). In South Korea, it is normative for companies to frequently use apologies and in conjunction with other crisis response strategies.

Although SCCT recommends organizations to apologize for a preventable crisis, South Korean companies predominantly use apologies regardless of the crisis type (Park and Ha, 2014; Kim and Lee, 2021). This is consistent with how East Asians apologize more often than Westerners in a face-threatening situation (Barnlund and Yoshioka, 1990; Park et al., 2005; Lee and Park, 2011) and crisis communication (Kim and Lee, 2021). For instance, South Korean companies may apologize for creating a disturbance and inconvenience even when they are not fully responsible for the crisis (Lim, 2020). Additionally, South Koreans tend to emulate others’ apologies more often than Americans (Park et al., 2005). Consequently, South Korean consumers are likely to expect an apology. Empirical evidence shows that apologies are highly effective in South Korea (Yoon and Choi, 2008; An et al., 2010). In other words, South Korean consumers may positively evaluate an apology, as it is consistent with their expectation, which reinforces other companies to model what worked previously.

Although crisis response strategies can be categorized into three groups, companies use them in combination (Kim et al., 2009). South Korean companies often combine apologies (i.e., saying ‘sorry’) with other crisis response strategies, even those from deny and diminish strategies (Yoon and Choi, 2008; Park and Ha, 2014; Lim, 2020). Kim and Yang (2012) content analysis of actual crisis response strategies used by South Korean organizations found that close to half of the apologies were insincere: the apologies included a justification of the crisis, appealed to sympathy, and excluded plans for corrective actions. Similarly, Kim and Lee (2021) analyzed 8 years of apologies used by South Korean and American organizations regarding cybersecurity breach. South Korean companies were more likely to include excuses, admittance of crisis responsibility, and display of concern and sympathy in their apologies. They were less likely to provide compensation and analytic accounts of the crisis compared to American companies. Lim (2020) also found that South Korean companies attack the accuser while apologizing for the crisis.

To better understand the effectiveness of crisis response messages that include apologies (which are prevalent yet diverse in South Korea), this study focuses on comparing two kinds of messages: one that includes an apology and a compensation, and the other that includes an apology and scapegoating. As the former includes two rebuilding strategies, it will be referred to as an accommodative apology (for simplicity). The latter will be referred to as a deny apology because scapegoating is an example of a deny strategy. The accommodative apology is more likely to convey a higher level of acceptance of crisis responsibility than the deny apology. An apology (i.e., saying sorry) conveys or at least implies acceptance of crisis responsibility, which can be enhanced when followed by compensation but reduced when followed by scapegoating. This is because both apology and compensation are rebuilding strategies according to SCCT (Coombs, 2010). As rebuilding strategies convey higher crisis responsibility compared to other types of response strategies, using two of them provides consistency in the message. Additionally, the compensation corroborates the sincerity of the apology. The accommodative apology shows the company’s willingness to own up to its faults and provide resolutions as it is willing to incur the financial burden of providing compensation (Coombs, 2006; Coombs and Holladay, 2008; Coombs et al., 2010). Consequently, accommodative apologies are likely to be perceived as sincere as it attempts to repair the relationship with the consumers (Bentley, 2018).

Compared to accommodative apologies, deny apologies convey less acknowledgement of crisis responsibility. While an apology shows (or at least implies) that the company is aware of its responsibility for the crisis, scapegoating discredits the accountability. Although the company is saying ‘sorry,’ it attempts to avoid taking responsibility for the crisis by attributing the crisis to a third party (Kim et al., 2004; Waller, 2007; Eisinger, 2011; Bentley, 2015). The authenticity of these apologies is easily questioned, making the consumers consider the company to be deceptive and manipulative (Compton, 2016). Therefore, deny apologies are less effective in reducing the public’s anger and negative sentiment (Lee and Chung, 2012).

Although the difference between accommodative and deny apologies is clear, companies still may have a difficult time deciding which apologies to use. As an accommodative apology demonstrates that the company is responsible for the crisis, it may have to handle financial repercussions for the crisis. Additionally, the company’s words may be used in lawsuits as justifications for why the company should compensate the victims (Patel and Reinsch, 2003). On the other hand, deny apologies leaves room for various interpretations of the company’s acknowledgment of the crisis responsibility (Pace et al., 2010). This enables the company to avoid the blame of the crisis while assuaging the consumers (to a certain extent) by apologizing (at least on the surface) for what has happened (Kim and Lee, 2021). When the crisis is detrimentally severe, an accommodative apology may be counterproductive; it provides self-supporting evidence that the company deserves punishment and blame for the crisis, which can further increase the perceived responsibility of the company (Schlenker, 1980; Sigal et al., 1988; O’Hara and Yarn, 2002). Therefore, scrutinizing which of the two apologies is more appropriate for whom in what situation enables the companies to make a more informed decision.

### Understanding appropriateness of apologies

When a company creates an apology for its consumers, the value and utility of the message depend on the perception of the receivers. Assuming that certain crisis response strategies are effective in restoring the damaged reputation because the company’s responsibility for the crisis matches the level of concern they express overlooks (1) the communicative process of sense-making, (2) the role of the message receiver in a communication, and (3) the mechanism that explains the effectiveness. The following elaborates on what it means for an apology to be appropriate, how it relates to restoring the damaged reputation, and how CRT helps us better understand the mechanism of appropriate apologies.

The appropriateness of an apology refers to the consumer’s perception of how well the company communicated to remedy the relationship with the consumer, which may have been damaged by the crisis. More specifically, SCCT literature suggests that the appropriateness of an apology comprises the consumer’s perception of its acceptability, sincerity, and effectiveness. First, the consumers are likely to accept apologies that they are satisfied with. In previous research (e.g., Coombs and Holladay, 2012; Bentley, 2018), appropriateness was operationalized as the consumer’s perceived acceptability of the apology. Second, the perceived sincerity of the apology is positively related to the appropriateness (e.g., Choi and Chung, 2013; Ebesu Hubbard et al., 2013; Lwin et al., 2017). Third, the effectiveness refers to how well the apology is anticipated to reduce the reputational damage of the company (Choi and Chung, 2013) or increase the stakeholder’s purchase intention (Lwin et al., 2017). As one of the goals of the apology is to yield a favorable evaluation and behavior from the consumer, the perceived effectiveness is the other pivotal aspect of the appropriateness. Considering the difference between the two apology types as mentioned above, the accommodative apology will be more appropriate than the deny apology.

The perceived appropriateness of the apology is positively related to the company’s reputation. Because a crisis directly and indirectly affects the product and services of the company, it negatively affects the consumer’s evaluation of the company (Dutta and Pullig, 2011). The consumer’s evaluation will remain negative unless there is evidence to believe otherwise. Crisis response messages are opportunities for companies to persuade consumers to rethink their evaluation. Therefore, companies can restore the damaged reputation by providing an apology that is appropriate (Choi and Chung, 2013). To examine the effect of apology type on the consumer’s perceived appropriateness and, consequently, on the company’s reputation, the following hypotheses are advanced and a research question is asked.

*H1*: The South Korean consumers will perceive the accommodative apology to be more appropriate than the deny apology.

*H2*: As the perceived appropriateness of the apology increases, the South Korean consumers will perceive the company’s overall reputation to be more positive.

RQ1: Will the apology type have a direct effect on the South Korean consumer’s perception of the company’s reputation above and beyond the mediation of appropriateness?

### CRT

Although apologies are inherently communicative acts, the communication between companies and consumers is seldomly examined from the perspective of meaning-making and sense-making (Weder et al., 2019). In this study, we use CRT as the theoretical framework to better situate SCCT in the context of communication. CRT illustrates how communicative parties (i.e., companies and consumers) make a judgment about how much responsibility each of them bears to create a mutual understanding (Aune et al., 2005). From Grice (1989) perspective, the goal of communication is to establish a shared understanding and knowledge. In other words, when communicating, people have the responsibility and commitment to engage in collaborative efforts in achieving this goal (Geurts, 2019). This responsibility is referred to as communicative responsibility. In other words, a company has responsibility for crisis (i.e., crisis responsibility) and responsibility for communicating with consumers (i.e., communicative responsibility). Crisis responsibility is evident at the time of the crisis and related to the attribution of the crisis. Communicative responsibility is relevant when crafting the crisis response message and interacting with its receivers.

In the context of crisis communication, CRT predicts that consumers judge the appropriateness of the company’s apologies based on the relative level of communicative responsibility. In other words, for consumers who perceive the company to bear a high level of communicative responsibility in helping the consumers under the crisis situation, they expect the company to use a message that adheres to their expectations. When the apology successfully helps these consumers to understand the crisis, the apology is perceived to be appropriate. In general, the consumers expect the company to have a higher communicative responsibility. According to Dibble (2012), the asymmetry in communicative responsibility is evident because the message creator (i.e., the company) has more communicative responsibility than the receiver (i.e., the consumers) when delivering bad news (i.e., the crisis). Furthermore, a crisis creates a state of uncertainty which consumers want to resolve (Coombs, 2000). As a result, they seek explanations for the cause of the crisis (Coombs et al., 2010). The crisis response messages that include explanations of the crisis are perceived to be appropriate (Dulaney and Gunn, 2018). According to Coombs (2007b), refusing to share information about the crisis with the consumer is unethical: companies have the obligation to protect the consumers who are vulnerable to the crisis.

**Figure 1:**
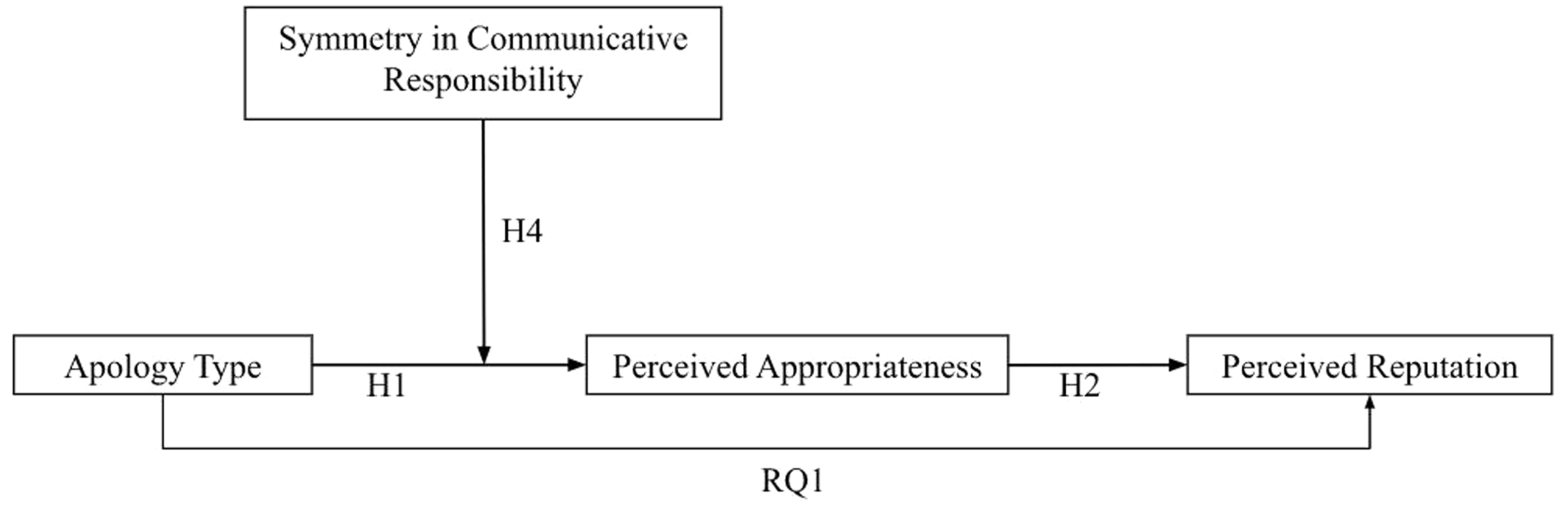
The hypothesized mediated moderation effect.

As the evaluation of communicative responsibility is based on subjective perception, there will be individual variations. For those who consider the company to have much more communicative responsibility than the consumers, they expect the company to devote much more effort to help them understand the situation. This devotion may be better reflected in an accommodative apology than the deny apology. When the company is blaming someone else for the crisis, the consumers are likely to perceive the whole message as insincere and unacceptable. As the authenticity of the message is questioned in a deny apology, consumers may consider the company to be deceptive and manipulative (Compton, 2016). Consequently, consumers doubt the company’s attribution of the crisis and the credibility of the company’s scapegoat, which prevents them from having an accurate sense of the crisis (Bonazzi, 1983). Thus, the difference in the appropriateness of the two apologies will be greater for consumers who perceive a larger asymmetry in the communicative responsibility (i.e., the company having a higher communicative responsibility than the consumers). In other words, the consumer’s perceived symmetry in communicative responsibility will moderate the relationship between apology type and appropriateness. Two hypotheses are advanced to examine these predictions (see Figure 1 for the conceptualized mediated moderation model):

*H3*: The South Korean consumers will perceive the communicative responsibility to be asymmetrical: the company has a higher responsibility than them.

*H4*: The difference between the perceived appropriateness of the accommodative and the deny apology will be greater for South Korean consumers who perceive the company to have a higher communicative responsibility than them.

## Materials and methods

### Participants and procedure

A total of 392 Koreans were recruited from a South Korean online survey company’s nation-wide panel^1^. Around half of them were women (49.48%), and their average age was 44.58 (*SD* = 13.18). Most of the participants received university-level or higher education (*n* = 334, 85%) and were working full-time (*n* = 244, 62%). The median interval for the participant’s monthly income was between two and three million won, which is consistent with the national median (Korean Statistical Information Service, 2021).

The participants first read that the seat belts of the car they recently purchased were malfunctioning for some time. They then read a news article that reported a traffic accident which caused severe casualties due to the malfunctioning of the seat belts. The same company the participants purchased their cars from also produced the cars mentioned in the news reports. We used a hypothetical company in this study to control for participant’s existing attitudes and prior experience. We chose an automobile company because numerous companies in this industry (e.g., BMW, Mercedes-Benz, Toyota, and Volkswagen) responded to crises using apologies (Ihlen, 2002; Hearit and Courtright, 2003; Choi and Chung, 2013; Trefis Team, 2016; Nam, 2018). Seat belt malfunction was chosen as automobile crises in South Korea are often related to defects in their products (including those related to the safety of the passengers; Min and Choi, 2015; Transportation Traffic Safety Team, 2016). The news article and vignettes are validated by Im et al. (2021).

To examine the appropriateness of different apologies, we randomly allocated participants to one of two groups. These groups read different vignettes that contained the crisis response messages of the company. In both conditions, the vignette included the company’s apology (i.e., “We apologize for the incident regarding the seat belt malfunction”) and a detailed account of the crisis (i.e., when and where the traffic accident happened). In the accommodative apology condition, the company stated that they will provide compensation for the victims. For the deny apology condition, the company scapegoated the crisis to the subcontractor who provides the necessary components of the seat belt. The participants then evaluated the appropriateness of the apology, the communicative responsibilities and the company’s reputation.

### Measures

We measured the communicative responsibility of the company and the participants, each with three items on a 5-point Likert scale (Aune et al., 2005). For instance, “given the context of this incident, it is mostly the company’s responsibility to make sure that the consumers understand the situation” and “it is appropriate, in this context, that the company works harder than the consumers to make certain that they understand this incident” are examples of items that measured the participant’s perception of the company’s communicative responsibility. To measure the participant’s perception of their own communicative responsibility, ‘consumers’ was replaced with ‘company’ and vice versa. The items were reliable (Cronbach’s α = 0.81 for both communicative responsibilities). We computed the mean scores for both communicative responsibilities.

The symmetry in communicative responsibility was computed by subtracting the mean score of the participant’s communicative responsibility from the mean score of the company’s communicative responsibility. A positive score indicates an asymmetry where the participants consider the company to have a higher degree of communicative responsibility than themselves. 0 indicates a perfect symmetry where the participant perceives the company to have the equal amount of burden in creating a mutual understanding about the crisis. A negative score indicates that the participants consider the company to have lower communicative responsibility than themselves.

The appropriateness of an apology was measured with 12 items on a 5-point Likert scale related to the perceived sincerity, acceptability, and effectiveness of the apology (Kim, 2006). A principal component analysis indicated that the 12 items were loaded on a single factor, explaining 79% of the variance. The 12 items were reliable (Cronbach’s α = 0.98). We computed the means score across the 12 items.

We measured the company’s reputation by modifying the five items on a 5-point Likert scale used in the study by Coombs and Holladay (2002) to fit the context of this study. For example, “the company is concerned with the well-being of the public” and “the organization is basically dishonest.” The Cronbach’s α was 0.87, indicating that the five items were reliable. We computed the mean score across these items.

## Results

### Manipulation check

This study conceptualized the two apologies to be different in the amount of crisis responsibility the company acknowledges. We used three items on a 5-point Likert scale to measure if the participants perceived the accommodative apology to exhibit a higher level of crisis responsibility acceptance than the deny apology (Coombs and Holladay, 2008). These items were reliable (Cronbach’s α = 0.94). The results of independent sample *t-*test indicates that the participants perceived the company to have accepted more responsibility of the crisis after reading the accommodative apology (*M* = 3.87, *SD* = 0.73) compared to the deny apology (*M* = 2.22, *SD* = 0.92), *t* (390) = 19.62, *p* < 0.001.

### Testing the symmetry in communicative responsibility

To examine if the participants perceived the company to have a higher communicative responsibility than the consumers in general (H3), we conducted a one-sample *t*-test that compared the symmetry in communicative responsibility with the test value of 0. The results indicated that there was an asymmetry in the communicative responsibility (*M* = 1.49, *SD* = 1.18), *t* (391) = 25.13, *p* < 0.001. Most of the participants (*n* = 325, 82.91%) perceived the communicative responsibility of the company to be higher than that of consumers. While 36 of them (9.18%) considered a perfect symmetry in communicative responsibility, there were some participants (*n* = 31, 7.91%) who perceived the consumers to have a higher burden of understanding what happened in the traffic accident compared to the company. Thus, the data was consistent with H3, but also indicates that there are individual differences in the assessment of communicative responsibility.

### Process analysis

To test and answer *H*1, *H*2, *H*4, and RQ1, a process analysis was conducted. According to Hayes (2018), the process analysis examines mediation and moderation effects in a single coherent model. In this study, model 8 was used to analyze the predicted relationships between the variables as illustrated in Figure 1. This model shows the effect of apology type on the reputation of the company to be mediated by the perceived appropriateness of the apology. In addition, the relationship between apology type and its appropriateness is moderated by the symmetry in communicative responsibility. To prepare for the process analysis, the apology type was dummy coded: the accommodative apology is 0, and the deny apology is 1. The moderator, symmetry in communicative responsibility, was mean-centered. To examine the interaction effect of apology type and the moderator, we selected a pick-a-point approach (16th, 50th, and 84th percentile of the moderator; Hayes, 2018).

**Figure 2:**
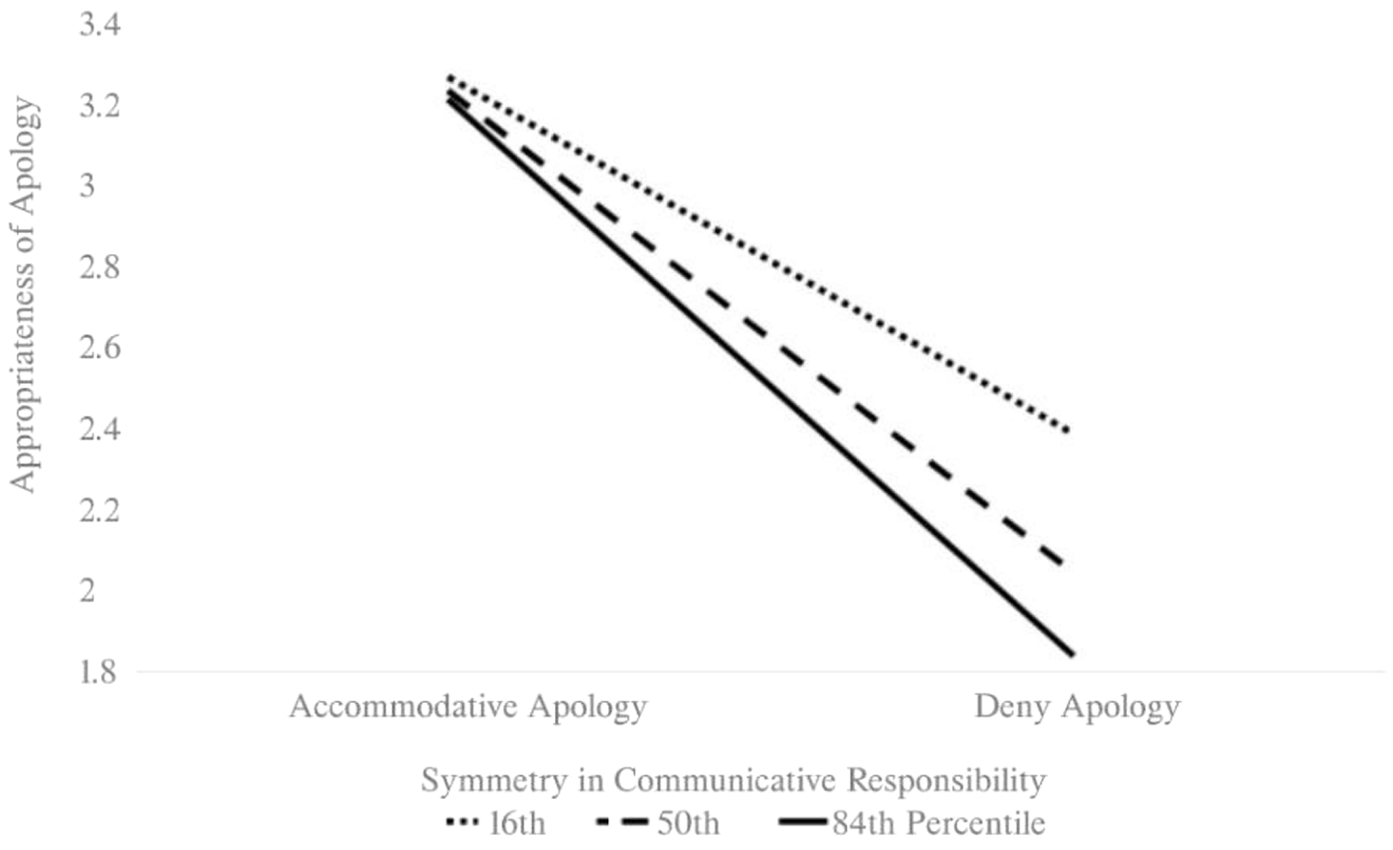
Simple slope analysis of signification moderation effect. The line graph shows the change in the effect of the apology type on its appropriateness on the three levels of symmetry in the communicative responsibility. The higher the percentile, the participants attributed more communicative responsibility to the company instead of the consumers.

The appropriateness of the apology was significantly related to the apology type and symmetry in communicative responsibility, *F* (3, 388) = 95.29, *p* < 0.001, *R*^2^ = 0.42 (see Table 1). The participants perceived the accommodative apology to be more appropriate than the deny apology (*B* = −1.16). The significant interaction effect between apology type and symmetry in communicative responsibility indicates that the difference between the two apologies varies across individuals (*B* = −0.19). The pick-a-point approach shows that the participants who perceived the company to have a relatively high communicative responsibility (i.e., the 84th percentile of the symmetry in communicative responsibility) finds deny apology to be much more inappropriate compared to those who put less communicative burden on the company (see Table 2; Figure 2). Therefore, the data was consistent with *H*1 and *H*4.

**Table 1:** Summary of process model.

	Dependent variable					
	Appropriateness			Reputation		
	*B*	*SE*	*t*	*B*	*SE*	*t*
Apology type^1^	−1.16	0.07	−16.29***	0.03	0.06	0.44
Appropriateness of apology	-	-	-	0.7	0.03	21.59***
Symmetry in communicative responsibility	−0.02	0.04	−0.48	−0.03	0.03	−1.09
Apology type × Symmetry in communicative responsibility	−0.19	0.06	−3.05**	<0.01	0.04	0.11
Constant	3.24	0.05	63.80***	0.94	0.11	8.57***
	*R^2^* = 0.42 (3, 388) = 95.29***			*R^2^* = 0.67 (4, 387) = 200.30***		

***p < 0.001, **p < 0.01, *p < 0.05. ^1^Dummy coded with the accommodative apology as the referent group.

**Table 2:** The moderating effects of symmetry in communicative responsibility.

	Apology type → Appropriateness			
	*B*	*SE*	*t*	95% bootstrap CI^1^
Symmetry in communicative responsibility				
−1.49 (16th percentile)	−0.88	0.2	−7.67***	−1.11 to −0.66
0.17 (50th percentile)	−1.19	0.07	−16.56***	−1.34 to −1.05
1.17 (84th percentile)	−1.38	0.1	−13.70***	−1.58 to −1.18
	**Apology type → Reputation**			
	***B***	***SE***	***t***	**95% CI**
Symmetry in communicative responsibility				
−1.49 (16th percentile)	0.02	0.08	0.25	−0.14 to 0.17
0.17 (50th percentile)	0.03	0.06	0.44	−0.09 to 0.14
1.17 (84th percentile)	0.03	0.03	0.4	−0.12 to 0.18

***p < 0.001, **p < 0.01, *p < 0.05. ^1^5000 bootstrapping iterations to calculate 95% confidence interval.

Regarding H2, the process model examining the effects of apology type, appropriateness of the apology, and symmetry in communicative responsibility on the reputation of the company was significant, *F* (4, 387) = 200.30, *p* < 0.001, *R*^2^ = 0.67. The results indicated that the appropriateness of the apology was positively related to the overall reputation (*B* = 0.70). However, the types of apology, communicative responsibilities, and their interaction were not significantly related to the reputation. Thus, the data was consistent with H2.

The direct effect analysis of the process model answers RQ1. As shown in Table 2, the direct effect was statistically insignificant on all three levels of the moderator (i.e., 16th, 50th, and 84th percentile of symmetry in communicative responsibility). This indicates that the appropriateness of the apology fully mediated the relationship between apology type and perceived reputation. Consequently, the relationship between apology type and reputation is a mediated moderation (see Muller et al., 2005).

## Discussion

This study examined the mediated moderation effect of apology type on the reputation of the automobile company in the context of crisis communication. The results of the process analysis indicated that an apology that includes compensation was more appropriate than those used with scapegoating. The perceived appropriateness was positively related to the perceived reputation of the company. There were individual differences in the perceived appropriateness of the apologies, which were explained by CRT. Compared to the appropriateness of the accommodative apology, the appropriateness of the deny apology varied depending on the individual’s perceived symmetry of communicative responsibility. For participants who attributed more communicative responsibility to the company, the deny apology was considered as less appropriate.

This study contributes to what Coombs and Holladay (2002) have recommended: we need more research on the mechanism behind the crisis response strategies, especially from the perspective of the message receivers. The mediated moderation effect examined in this research investigated the mechanism behind the relationship between apologies and the company’s reputation (i.e., the mediation) and the condition in which the relationship varies (i.e., moderator; Baron and Kenny, 1986). Consistent with the literature, the findings indicated that the composition of an apology is related to its appropriateness and, subsequently, the reputation of the company. According to Pace et al. (2010), the consumers may not consider the company to be acknowledging its crisis responsibility when it simply says, “we apologize for this situation.” For this reason, an apology needs to be complemented by other crisis response strategies, such as compensation, to validate its authenticity (Bentley, 2018). The consumers may consider the accommodative apologies to demonstrate the company’s willingness to take responsibility for the crisis and actively remedy the consequences of the crisis. When the company takes responsible actions instead of blaming others, the consumers are likely to see it positively because the apology conveys its willingness to learn from the mistake to prevent future recurrences (Schultz et al., 2011; Yuan et al., 2016). On the other hand, the deny apology is less appropriate and leads to less positive reputational evaluation from the consumers because it can make the consumers doubt the sincerity of the provided crisis response message. When the consumers believe that the company is not providing accurate and sufficient information about the crisis, consumers perceive the company to be less transparent (Coombs, 2007b). In other words, the consumer’s negative evaluation of the apology spills over to the company’s reputation.

Consumers do not always perceive or interpret the crisis response messages as intended by the company (Choi and Chung, 2013). This may be due to the different expectations that arise from idiosyncratic evaluations of communicative responsibility. For the participants who put more burden on the company to explain the crisis, the appropriateness of the deny apology decreases even more. As they expect more from the company to provide accurate and proper information, the negative violation of the deny apology is greater for them (Burgoon, 2015). Therefore, examining crisis communication from the perspective of collaborative meaning-making (as CRT suggests) helps us better understand the individual differences within the message receivers.

Although we examined two types of apologies that are likely to be used by South Korean companies in practice, the results should not be interpreted as ‘always use an accommodative apology because it is better at managing the reputation of the company.’ The company needs to weigh the difference in the appropriateness and potential repercussions of the apologies. Considering the different factors that affects assessment of communicative responsibility (Aune et al., 2005), a deny apology may be relatively more appropriate when the crisis is straightforward and simple for the consumers to understand, when uncertainty is considered a virtue in the consumers’ culture, and when the company is clearly the victim (i.e., the consumers agree that the company also does not know when or how the crisis happened). In cases where the company clearly has disproportionately high communicative responsibility, an accommodative apology may be more beneficial despite the financial liabilities (Hearit, 2006; Coombs, 2007a). When the company fails to appease the disappointed consumers, it may need to apologize again (Compton, 2016), and face a reduction in market share and consumer loyalty (Blodgett et al., 1995). Thus, in the long run, a well-received apology can successfully minimize the company’s financial costs (Tax et al., 1998).

We should consider the South Korean context when interpreting the findings. This study focused specifically on apologies, as the majority of South Korean companies (78.65%) used them regardless of the crisis situation (Park and Ha, 2014). According to Claeys and Schwarz (2016), apologies may be perceived as less sincere in cultures where apologies are more frequently used. Although South Korean consumers expect apologies, they may be easily skeptical of the sincerity when there is evidence to do so (e.g., scapegoating). Additionally, the collectivistic culture in South Korea encourages collective responsibility. South Koreans are likely to be angry and perceive the company negatively when it attributed the crisis to a single employee instead of taking responsibility as an organizational entity (An et al., 2010). The expectation of collective responsibility may also apply to crises that are caused by the subcontractors. Consequently, the normative and cultural context may explain South Koreans’ sensitivity to deny apologies. The generalization of this study’s findings may be limited to other cultures that emphasize collective responsibility (e.g., Japan; Barkley, 2020). As companies in the United States tend to give excuses more frequently than South Korean companies (Kim and Lee, 2021), the inappropriateness of deny apologies may be less severe.

### Limitations and future directions

Although the South Korean context is noteworthy as its companies are being globally recognized and economically impactful, this study is limited to a single cultural context. A cross-cultural study is needed to (a) better understand to what extent the findings of a single culture can be generalized, and (b) examine what specific cultural differences or similarities explain the results. We encourage future researchers to investigate the relationships between cultural variables (e.g., collectivism/individualism and high/low context), crisis attribution, communicative responsibility, and crisis message appraisal.

The scope of this research is limited to a single crisis situation (i.e., product malfunction). Therefore, the given situation may be categorized as either an accidental or a preventable crisis depending on how the crisis is described. For instance, if an unexpected equipment failure caused the product malfunction, the given situation can be categorized into the accidental cluster. However, if the product malfunction was intended by a member of the company, it is a preventable crisis. According to Coombs and Holladay (2002), crisis type should be evaluated before devising a crisis response strategy. This is because each crisis cluster attributes a similar amount of crisis responsibility to the company, which enables it to use a similar response for various crises within a cluster. Additionally, the consumer may find an apology to be insincere if the company is highly accountable for the crisis (Lwin et al., 2017). In this case, the difference between the appropriateness of the two apologies may be smaller than what was examined in this study. Additionally, considering the cultural differences in crisis attribution, intersecting variations in culture and in crisis situation can push the envelope of crisis communication research.

In respect to CRT, the asymmetry in the communicative responsibility may change depending on the company’s responsibility for the crisis. For instance, Dulek and Campbell (2015) found that CEOs may strategically use indirect language and avoid explicit explanations when communicating about the unexpected collapse of the financial market with the stakeholders. Because the incident was unanticipated, the CEOs have low crisis responsibility. Additionally, the communicative responsibilities of the CEO and the stakeholders are symmetrical, as neither of them are fully aware of what happened and what will happen next. Therefore, being strategically vague in communication is justified. Thus, it is recommended for future research to evaluate how communicative responsibility changes as the company’s crisis responsibility changes.

Future researchers can also examine the effect of a company’s performance history on the appropriateness of the crisis response. It is possible for the consumers to perceive an accommodative apology as insincere if the company has a long history of repeated misdeeds. The seemingly appropriate apology (i.e., accommodative apology) may backfire when the consumers do not trust the company. By utilizing an existing organization, instead of a hypothetical one, the impact of the company’s crisis history can be examined. Therefore, future research may benefit from conducting case studies.

## Data availability statement

The raw data supporting the conclusions of this article will be made available by the authors, without undue reservation.

## Ethics statement

Ethical review and approval was not required for the study on human participants in accordance with the local legislation and institutional requirements. The patients/participants provided their written informed consent to participate in this study.

## Author contributions

All authors listed have made a substantial, direct, and intellectual contribution to the work and approved it for publication.

## Funding

This work was partially funded by the School of Media and Communication at Korea University.

## Conflict of interest

The authors declare that the research was conducted in the absence of any commercial or financial relationships that could be construed as a potential conflict of interest.

## Publisher’s note

All claims expressed in this article are solely those of the authors and do not necessarily represent those of their affiliated organizations, or those of the publisher, the editors and the reviewers. Any product that may be evaluated in this article, or claim that may be made by its manufacturer, is not guaranteed or endorsed by the publisher.

## Footnotes

^1^ https://www.embrain.com/eng/
